# An individual-based dataset of carbon and nitrogen isotopic data of *Callinectessapidus* in invaded Mediterranean waters

**DOI:** 10.3897/BDJ.10.e77516

**Published:** 2022-01-25

**Authors:** Cristina Di Muri, Ilaria Rosati, Roberta Bardelli, Lucrezia Cilenti, Daniel Li Veli, Silvia Falco, Salvatrice Vizzini, George Nikolaos Katselis, Kosmas Kevrekidis, Luka Glamuzina, Giorgio Mancinelli

**Affiliations:** 1 National Research Council (CNR), Research Institute on Terrestrial Ecosystems (IRET), Lecce, Italy National Research Council (CNR), Research Institute on Terrestrial Ecosystems (IRET) Lecce Italy; 2 LifeWatch ERIC, Seville, Spain LifeWatch ERIC Seville Spain; 3 University of Palermo, Palermo, Italy University of Palermo Palermo Italy; 4 National Research Council (CNR), Institute of Marine Biological Resources and Biotechnologies (IRBIM), Lesina, Italy National Research Council (CNR), Institute of Marine Biological Resources and Biotechnologies (IRBIM) Lesina Italy; 5 National Research Council (CNR), Institute of Marine Biological Resources and Biotechnologies (IRBIM), Ancona, Italy National Research Council (CNR), Institute of Marine Biological Resources and Biotechnologies (IRBIM) Ancona Italy; 6 University of Valencia, Valencia, Spain University of Valencia Valencia Spain; 7 National Interuniversity Consortium for Marine Sciences (CoNISMa), Roma, Italy National Interuniversity Consortium for Marine Sciences (CoNISMa) Roma Italy; 8 University of Patras, Mesologhi, Greece University of Patras Mesologhi Greece; 9 Decentralised Administration of Macedonia Thrace, Kalamaria, Greece Decentralised Administration of Macedonia Thrace Kalamaria Greece; 10 University of Dubrovnik, Dubrovnik, Croatia University of Dubrovnik Dubrovnik Croatia; 11 University of Salento, Lecce, Italy University of Salento Lecce Italy

**Keywords:** invasive species, Atlantic blue crab, transitional water, stable isotope, trophic position, isotopic niche

## Abstract

**Background:**

The characterisation of functional traits of non-indigenous and invasive species is crucial to assess their impact within invaded habitats. Successful biological invasions are often facilitated by the generalist diet of the invaders which can modify their trophic position and adapt to new ecosystems determining changes in their structure and functioning. Invasive crustaceans are an illustrative example of such mechanisms since their trophic habits can determine important ecological impacts on aquatic food webs. The Atlantic blue crab *Callinectessapidus* is currently established and considered invasive in the Mediterranean Sea where it has been recorded for the first time between 1947 and 1949. In the last decade, the blue crab colonised most of the eastern and central Mediterranean Sea and the Black Sea and it is currently widening its distribution towards the western region of the basin.

**New information:**

Stable isotope analysis is increasingly used to investigate the trophic habits of invasive marine species. Here, we collated individual measures of the blue crab δ^13^C and δ^15^N values and of its potential invertebrate prey into a geo-referenced dataset. The dataset includes 360 records with 236 isotopic values of the blue crab and 224 isotopic data of potential prey collected from five countries and 12 locations between 2014 and 2019. This dataset allows the estimation of the trophic position of the blue crab within a variety of invaded ecosystems, as well as advanced quantitative comparisons of the main features of its isotopic niche.

## Introduction

The concern about the impacts of non-indigenous species (NIS) has grown steeply over the past half-century (David et al. 2017), and a large amount of evidence suggests that NIS can alter the structure of natural communities and the integrity of ecosystems causing substantial ecological, economic, and cultural losses, especially in case they become invasive (Invasive Alien Species, IAS hereafter; Anton et al. 2019, see also Thomsen 2020). The impact of IAS is mediated by biotic interactions with the native communities and changes in the ecosystem processes including nutrient dynamics, fluxes of energy, and material cycling (Gallardo et al. 2016, Corrales et al. 2020). The monitoring and assessment of the impact of IAS and the prioritisation of adequate management actions are crucial steps to mitigate and/or limit the potential adverse effects of those species that are more likely to represent a serious threat to native ecosystems (Dick et al. 2017, Cuthbert et al. 2019).

The Atlantic blue crab (*Callinectessapidus* Rathbun, 1896) is considered one of the worst invasive species in the Mediterranean Sea owing to its impact on local biodiversity, fisheries, and aquaculture (Streftaris and Zenetos 2006). The Atlantic blue crab (hereafter blue crab) was first recorded in Europe at the start of the past century (Bouvier 1901) and appeared in the Mediterranean Sea between 1947 and 1949 (Giordani Soika 1951, Serbetis 1959). Over the last decade, the blue crab has spread almost ubiquitously in the eastern and central Mediterranean Sea and in the Black Sea, and it is currently widening its distribution towards the western sectors of the basin (Mancinelli et al. 2017a, Mancinelli et al. 2021).

The blue crab is an opportunistic omnivore, feeding on a variety of food sources from plants and detritus, to molluscs, arthropods (including conspecifics), polychaetes, and fish (Belgrad and Griffen 2016). Similar to other IAS, such trophic plasticity represents a key adaptive feature explaining the establishment success of the blue crab in non-native ecosystems and its impact on invaded food webs (Mancinelli et al. 2017b). However, while the crucial role played by the blue crab in regulating the structure and functioning of native food webs is well recognised, the number of studies investigating the trophic role of this invader in non-native ecosystems is remarkably lower (Mancinelli et al. 2017b, but see Kampouris et al. 2019 for a counter-example). In native coastal ecosystems, the blue crab acts as a keystone species by regulating the carbon cycle and prey/predator abundance through both bottom-up and top-down interactions (Altieri et al. 2012, Pugesek et al. 2013). Thus, understanding how the trophic ecology of this taxon shapes benthic food webs in invaded ecosystems is crucial for an accurate assessment of its impact.

Stable isotope analysis of δ^13^C and δ^15^N has become an increasingly popular methodology in studies focusing on food web structure and functioning. Specifically, δ^13^C can trace the flow of matter and nutrients from basal to higher trophic levels and δ^15^N can clarify trophic interactions (Fry 2006). In addition, even though species-specific physiological or metabolic factors can influence δ^15^N values (Tibbets et al. 2007; Doi et al. 2017), δ^15^N is commonly used to infer the species trophic position within food webs (Post 2002). In fact, as the trophic level increases, the nitrogen enriches predictably in its heavier isotope ^15^N due to the preferential excretion of the lighter isotope ^14^N (Minagawa and Wada 1984; Post 2002). Noticeably, the assessment of the effects of non-indigenous species on native communities is one of the first applications of stable isotope analysis in invasion ecology (Mitchell et al. 1996). Mancinelli and Vizzini (2015)reviewed the advantages and limitations of the method for identifying and quantifying the ecological impact of invasive species, clearly emphasising how the estimation of invasive species trophic position, using stable isotopes, can be successfully used for assessing direct predatory impacts, as well as community-scale effects on the whole trophic structure. For the blue crab, recent evidence indicated substantial variability in its trophic level across closely-located coastal ecosystems and size-related shifts in individual trophic position within invaded food webs (Mancinelli et al. 2016, Mancinelli et al. 2017b). Ultimately, these studies demonstrate the utility of stable isotope analysis in detecting changes in food web structure after the invasion; an advanced understanding of such trophic interactions can, in turn, help to predict and quantify the impact of biological invasions on aquatic food webs (Jackson et al. 2012).

## General description

### Purpose

This dataset collates available geo-referenced and individual-based isotopic values (δ^13^C and δ^15^N) of *C.sapidus* and its potential animal prey in Mediterranean waters. The isotopic values, included in the dataset, are expressed in delta notation (‰ deviation from atmospheric nitrogen and from Pee Dee Belemnite [PDB] limestone used as standards for N and C, respectively) and δ^15^N or δ^13^C = [(R_Sample_/R_Standard_) – 1] × 1000, where R = ^15^N/^14^N or ^13^C/^12^C. The analytical precision of measurements for all δ^13^C and δ^15^N values was 0.2‰ as calculated by the standard deviation of replicates of the internal standards. This dataset can be used for a variety of comparative analyses including the calculation of the trophic position of the crab and/or metrics and descriptors of its isotopic niche, and it was conceived as one of the input files for the Functional biogeography of invaders workflow of the LifeWatch ERIC Internal Joint Initiative. Specifically, the analytical workflow aims at identifying climatic predictors of the trophic position of two invasive crustaceans, i.e. the blue crab *C.sapidus* and the Louisiana crayfish *Procambarusclarkii*. For *P.clarkii*, the workflow runs on an aggregated dataset resolved at population scale, whereas for *C.sapidus*, two datasets can be used as input files to run the analyses: (i) an aggregated dataset at the population scale similar to the one built for *P.clarkii* and (ii) an individual-based dataset with isotopic values of single specimens as described in the present article. All datasets include isotopic information for potential prey to allow the estimation of the trophic position of the invasive species under analysis.

## Project description

### Title

LifeWatch ERIC Internal Joint Initiative - Functional biogeography of invaders: the case of two widely distributed omnivorous crustaceans (https://bit.ly/iji-crustaceans).

### Personnel

Cristina Di Muri, Giorgio Mancinelli, Ilaria Rosati, Lucia Vaira

### Study area description

Coastal and transitional areas of the Mediterranean Sea colonised by *C.sapidus*. The westernmost records are located in Spain, the northernmost in Croatia, the easternmost in Turkey, and the southernmost in Greece. The majority of records lie in Italy.

### Design description

The dataset contains geographical and temporal information on the sampling event including country, location, geographical coordinates, type of habitat, year, and month or season in which the sampling occurred. Biological features of the species included are also specified, such as the invasive or native nature of the species for each location and the prey-predator relationship. Such attributes, together with δ^13^C and δ^15^N values, can be used for downstream analyses including the calculation of the blue crab trophic position, which can be estimated using two different approaches. The first method estimates the trophic position using the following equation:

Trophic Position δ^15^N = (δ^15^N_Consumer_ - δ^15^N_Baseline_)/Δ^15^N + λ

This equation is a generalisation of the formula presented in Jepsen and Winemiller (2002), where δ^15^N_Consumer_ is the nitrogen isotopic value of the blue crab, Δ^15^N is the trophic level fractionation of δ^15^N, δ^15^N_Baseline_ and λ are the nitrogen isotopic value and the trophic level of the baseline species (e.g. for *Phorcusturbinatus*, λ = 2). Alternatively, the blue crab trophic position can be estimated using a Bayesian approach implemented in the R package tRophicPosition (Quezada‐Romegialli et al. 2018, R Core Team 2021). In this case, the original δ^13^C and δ^15^N values can be back-estimated using mean values, standard deviations, and sample size for each location and species and assuming a normal distribution.

## Sampling methods

### Study extent

The literature search for compiling this dataset ended on 31^st^ April 2021.

### Sampling description

The online platforms ISI Web of Science and Scopus were searched using multiple search criteria including the terms “*Callinectessapidus*” and “stable isotopes” in conjunction with “non-indigenous”, “alien”, “invasive”, “Mediterranean Sea”, and “Black Sea”. The results were integrated with those obtained by querying Google Scholar using the same search criteria, together with the corresponding terms in Spanish or Portuguese (e.g. “jaiba azul”, “cangrejo azul”, “siri azul”) in order to access additional literature published in languages other than English. Google Scholar search results were saved using the freeware Publish or Perish ver. 7.27.2849 (Harzing 2007).

### Quality control

Only records with defined locations whose accuracy was checked using Google Earth were included in the dataset; geographic coordinates were converted to decimal degrees when not originally specified as such. The taxonomic check was performed using the World Register of Marine Species.

### Step description

The blue crab preys preferentially on bivalves (Hines 2007); accordingly, this taxonomic group was chosen as a reference for the selection of baseline species included in the dataset. *M.galloprovincialis* was generally used in the dataset given the almost ubiquitous distribution in Mediterranean marine coastal waters. If not available, other bivalves and herbivorous gastropods occurring at the study sites were chosen. On only one occasion, the omnivorous polychaete (*Alittasuccinea*) was used as the baseline species. The trophic level of prey species was assigned, based on their trophic habits as follows: bivalves’ trophic position = 2 (filter feeders); gastropod *Phorcusturbinatus* trophic position = 2 (herbivore; Aslan and Polito 2021); polychaete *A.succinea* trophic position = 2.78 (omnivore; Jumars et al. 2015, Como et al. 2018). Both *M.galloprovincialis* and *Arcuatulasenhousia* are filter feeders and their diets mainly rely on phytoplankton and suspended particulate matter (Inoue and Yamamuro 2000, Ezgeta-Balić et al. 2014); hence, in agreement with the Sea Around Us database, we assumed for both taxa a trophic position = 2. However, it has been also repeatedly indicated that zooplankton can be included in the diet of *M.galloprovincialis* depending on local conditions, as well as on seasonal variations in resource availability (Ezgeta-Balić et al. 2012, Peharda et al. 2012). Our dataset is freely downloadable and the trophic position of *C.sapidus* prey can be modified according to the user's specific needs and/or if and when more updated information becomes available. Within our dataset a considerable variation in individual δ^15^N values occur for *M.galloprovincialis* as well as for other baseline species (i.e. *Phorcusturbinatus*). Such variation could be due to individual-scale differences in feeding habits, ontogeny, and metabolism (Tibbets et al. 2007, Doi et al. 2017). The user is, therefore, free to calculate the trophic position of *C.sapidus* by adopting, for example, the grand mean of the δ^15^N values or any other estimation of central tendency (e.g. mode or median). Alternatively, individual δ^15^N values of baseline taxa can be selected by eliminating the outliers or by choosing only a subset of the specimens included (e.g. the lower quartile of the isotopic distribution).

## Geographic coverage

### Description

**Figure 1.**
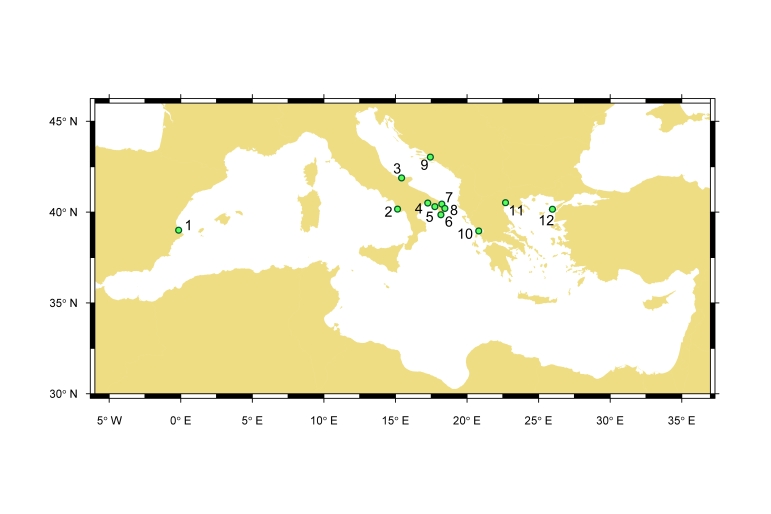
Map of the Mediterranean sites included in the dataset. The study sites represent coastal and transitional systems where established populations of *Callinectessapidus* were investigated.

The dataset gathers isotopic values of different Mediterranean areas colonised by *C.sapidus* including seven study sites in Italy, two study sites in Greece, and one study site in Croatia, Spain, and Turkey (Fig. 1, Table 1). The westernmost records are located in Spain (39.00494; -0.148602), the northernmost in Croatia (43.027423; 17.436056), the easternmost in Turkey (40.157275; 25.96473), and the southernmost in Greece (38.961748; 20.815487).

### Coordinates

38.96175 and 43.02742 Latitude; -0.1486 and 25.96473 Longitude.

## Taxonomic coverage

### Description

The dataset is a collection of individual-based isotopic values belonging to *C.sapidus* and its potential prey including: *P.turbinatus*, *A.succinea*, *Arcuatulasenhousia*, and *M.galloprovicialis*.

### Taxa included

**Table:** 

Rank	Scientific Name	Common Name
species	*Alittasuccinea*	Pile worm
species	*Arcuatulasenhousia*	Asian date mussel
species	*Callinectessapidus*	Blue crab
species	*Mytilusgalloprovincialis*	Mediterranean mussel
species	*Phorcusturbinatus*	Turbinate monodont

## Usage licence

### Usage licence

Creative Commons Public Domain Waiver (CC-Zero)

### IP rights notes

This work is licensed under a Creative Commons Attribution (CC-BY) 4.0 Licence.

## Data resources

### Data package title

An individual-based dataset of carbon and nitrogen isotopic data of *Callinectessapidus* in invaded Mediterranean waters.

### Resource link

https://doi.org/10.48372/9CD2907F-8F44-4DF6-9070-6B21E1991B94

### Number of data sets

1

### Data set 1.

#### Data set name

An individual-based dataset of carbon and nitrogen isotopic data of *Callinectessapidus* in invaded Mediterranean waters.

#### Data format

csv

#### Number of columns

13

#### Download URL

https://dataportal.lifewatchitaly.eu/view/urn%3Auuid%3Ae869c2cc-2011-461d-99cc-8d6d642b1624

#### Description

A description of the dataset format is provided below. The dataset attributes were labelled using standard glossaries harvested from Darwin Core, LifeWatch ERIC Ecoportal, and NERC Vocabulary Server.

**Data set 1.** 

Column label	Column description
catalogNumber	An identifier (preferably unique) for the record within the dataset or collection.
country	The name of the country or major administrative unit in which the Location occurs.
locality	The specific description of the place.
habitat	A category or description of the habitat in which the Event occurred.
eventDate	The date-time or interval during which an Event occurred.
decimalLatitude	The geographic latitude (in decimal degrees, using the spatial reference system given in geodeticDatum) of the geographic centre of a Location. Positive values are north of the Equator, negative values are south of it.
decimalLongitude	The geographic longitude (in decimal degrees, using the spatial reference system given in geodeticDatum) of the geographic centre of a Location. Positive values are east of the Greenwich Meridian, negative values are west of it.
establishmentMeans	Statement about whether an organism or organisms have been introduced to a given place and time through the direct or indirect activity of modern humans (https://dwc.tdwg.org/em/#dwcem_e).
scientificName	The full scientific name, with authorship and date information, if known. When forming part of an Identification, this should be the name in lowest level taxonomic rank that can be determined. This term should not contain identification qualifications, which should instead be supplied in the IdentificationQualifier term.
trophicRole	Statement specifying whether the species is a predator or a prey.
carbon-13	A value of the isotope of the chemical element carbon, expressed in permil (‰).
nitrogen-15	A value of the isotope of the chemical element nitrogen, expressed in permil (‰).
trophicLevel	Any of the feeding levels through which the passage of energy through an ecosystem proceeds; examples are photosynthetic plants, herbivorous animals, and microorganisms of decay.

## Figures and Tables

**Table 1.** List of locations included in the dataset with names, countries, ecosystem types, and years in which the sampling events occurred. The last column states the source from which the information was extracted.

**Location ID**	**Location name**	**Country**	**Habitat**	**Sampling year**	**Reference**
1	Gandia	Spain	Estuary	2016	De Giorgi et al. (2022)
2	Alento	Italy	Estuary	2019	Li Veli et al. (2022)
3	Lesina	Italy	Lagoon	2016	De Giorgi et al. (2022)
4	Mar Piccolo	Italy	Lagoon	2014	Mancinelli et al. (2017b)
5	Torre Colimena	Italy	Lagoon	2014	Mancinelli et al. (2017b)
6	Spunderati	Italy	Lagoon	2014	Mancinelli et al. (2017b)
7	Acquatina	Italy	Lagoon	2016	Mancinelli et al. (2017b)
8	Alimini	Italy	Lagoon	2014	Mancinelli et al. (2017b)
9	Parila	Croatia	Lagoon	2015	Mancinelli et al. (2016)
10	Pogonitsa	Greece	Lagoon	2016	De Giorgi et al. (2022)
11	Loudias	Greece	Coastal	2016	De Giorgi et al. (2022)
12	Gökçeada	Turkey	Lagoon	2017	Aslan and Polito (2021)
